# Perceived workload and teaching innovative behavior in university teachers: parallel mediation by need for cognitive closure and thriving at work, gender multi-group

**DOI:** 10.3389/fpsyg.2026.1837443

**Published:** 2026-09-14

**Authors:** Man Chen, Ling Zhang

**Affiliations:** 1 Research Center for Continuing Education and Digital Applications, Henan Open University, Zhengzhou, Henan, China; 2 Research Center for Collaborative Innovation in Open and Vocational Education, Henan Open University, Zhengzhou, Henan, China

**Keywords:** need for cognitive closure, perceived workload, teaching innovative behavior, thriving at work, university teachers

## Abstract

**Background:**

In the context of increasing demands in higher education, university teachers are expected to engage in innovative teaching while managing growing workloads. Drawing on conservation of resources theory, this study examined the association between perceived workload and teaching innovative behavior among university teachers, as well as the mediating roles of need for cognitive closure and thriving at work. Although perceived workload has been associated with innovative behavior, limited research has simultaneously considered the cognitive and psychological processes underlying this association in higher education.

**Methods:**

Data were collected from 634 university teachers through a cross-sectional questionnaire survey using convenience sampling and analyzed using partial least squares structural equation modeling.

**Results:**

The results showed that perceived workload was negatively associated with teaching innovative behavior. Need for cognitive closure and thriving at work both significantly mediated this association. Specifically, higher perceived workload was associated with a stronger need for cognitive closure, which was subsequently associated with lower teaching innovative behavior. Perceived workload was negatively related to thriving at work, which was, in turn, positively related to teaching innovative behavior. The negative relationship between perceived workload and teaching innovative behavior was also more pronounced among female teachers than among their male counterparts.

**Conclusion:**

These findings identify two conceptually distinct but directionally consistent indirect pathways linking perceived workload to teaching innovative behavior. This study extends the application of conservation of resources theory to university teaching and highlights the importance of managing teachers’ workload and protecting the cognitive and psychological resources required for innovative teaching practices.

## 1 Introduction

Digitalization and artificial intelligence are reshaping teaching and learning in higher education. The widespread use of multimedia technologies and online learning resources, together with the growing emphasis on cultivating students’ innovative capacities, requires university teachers to continually update their teaching content, methods, and assessment practices. Consequently, teaching innovative behavior (TIB) has become increasingly important for improving teaching quality and supporting the development of higher education.

TIB refers to the generation and application of novel, useful ideas in teaching and represents an important indicator of higher education quality and competitiveness (Cheng and Xu, 2025). However, teaching innovation requires substantial investments of time, energy, attention, and cognitive effort (Zhang, 2025). University teachers must simultaneously manage teaching, research, student supervision, administrative duties, and continuously changing technological requirements, which may contribute to a high perceived workload (Liu et al., 2023). Under such conditions, teachers may have fewer resources available for developing and implementing innovative teaching practices.

Individual and environmental factors both influence innovative behavior. Among them, external environmental factors include perceived workload, overqualification, organizational identification and distributed leadership (Kim, 2026; Ma and Zhang, 2025; Xiang et al., 2024; Zhang X. X. et al., 2024). Perceived workload reflects teachers’ assessments of task volume and difficulty and is negatively associated with their intrinsic motivation and innovative behavior (Li et al., 2025b; Wang et al., 2025). Intra-individual factors include need for cognitive closure (NFCC), thriving at work (TW), and teaching effectiveness (Ortega-Martín et al., 2021; Wang et al., 2022; Zhang X. B. et al., 2024). NFCC reflects a preference for definite answers and an aversion to ambiguity, which may restrict the exploration of alternative teaching approaches (Ortega-Martín et al., 2021). TW, characterized by vitality and learning, represents a positive psychological state that may support teachers’ engagement in innovative teaching practices (Christensen-Salem et al., 2021). Although previous studies have separately examined the relationships of perceived workload, NFCC, and TW with creativity or other innovation-related outcomes, this evidence remains fragmented and is insufficient to explain the specific psychological pathways underlying the association between perceived workload and university teachers’ TIB. In particular, it remains unclear whether higher perceived workload is associated with a stronger preference for certainty and reduced teaching exploration, with insufficient resources, or with both processes simultaneously. Therefore, whether NFCC and TW constitute two distinct yet complementary indirect pathways between perceived workload and TIB remains to be systematically examined. The current study uses conservation of resources (COR) theory to create a parallel mediation model that investigates the relationship between perceived workload and TIB while concurrently evaluating the mediating functions of NFCC and TW in order to address this unresolved issue (Hobfoll, 1989). Specifically, the NFCC pathway captures the potential process through which teachers conserve their remaining cognitive resources by seeking certainty and reducing exploration, whereas the TW pathway examines whether teachers possess sufficient vitality and learning resources to engage in teaching innovation. By comparing and integrating these two indirect pathways, this study aims to offer a more nuanced explanation of the association between perceived workload and university teachers’ TIB from the perspectives of cognitive resource conservation responses and psychological resource availability.

### 1.1 Theoretical framework

According to COR theory, people work hard to preserve important resources including time, energy, focus, and psychological capability (Hobfoll, 1989). When situational demands surpass these resources, they become stressed. Under sustained resource pressure, individuals may respond not only by reducing their investment in resource-intensive activities but also by adopting strategies that protect their remaining resources. In the context of higher education, perceived workload may threaten university teachers’ limited cognitive and psychological resources, whereas TIB requires sustained attention, cognitive flexibility, vitality, learning, and continued effort. Perceived workload may therefore be associated with lower TIB through multiple resource-related processes.

NFCC represents a cognitive resource-conservation tendency within this framework. When teachers perceive that their workload exceeds the cognitive resources available to manage it, they may prefer clear, stable, and predictable information, rely more heavily on familiar teaching routines, and seek rapid closure rather than continue considering multiple alternatives (Büning et al., 2021). Such a response may help teachers reduce cognitive effort and manage uncertainty in the short term. However, it may also narrow their search for alternative teaching approaches and reduce their willingness to experiment with unfamiliar ideas. Because TIB requires openness, flexibility, tolerance of uncertainty, and the continued exploration of new teaching methods, a stronger NFCC may inhibit teachers’ engagement in innovative teaching practices (Wronska et al., 2019).

TW, by contrast, stands for the accessibility of constructive psychological and developmental resources. It is defined by the combined sensation of learning and vitality: learning is the feeling of gaining and using new information and abilities, while vitality is the energy available for continuous participation (Spreitzer et al., 2005). Perceived workload may consume teachers’ time, energy, and attention, leaving fewer resources available for professional learning and reducing the vitality needed to develop and implement innovative teaching ideas (Goh et al., 2022). Lower TW may consequently limit teachers’ capacity to explore new teaching approaches, acquire relevant knowledge, and persist in the implementation of teaching reforms. Therefore, the pathway through TW reflects the depletion of the psychological and developmental resources required to support innovative teaching.

The two mediating pathways are conceptually distinct yet complementary. NFCC reflects how teachers respond to workload pressure by seeking certainty, reducing the exploration of alternatives, and limiting further cognitive investment to conserve their remaining cognitive resources (Büning et al., 2021). TW, by contrast, reflects whether teachers retain the vitality and learning resources required for TIB (Goh et al., 2022). The former represents a cognitive resource conservation response under resource pressure, whereas the latter indicates the availability of the psychological and developmental resources necessary for innovation. Teachers may experience either process independently or both simultaneously; therefore, the two mechanisms are not interchangeable. Compared with earlier research that has mainly focused on the direct association between perceived workload and innovative behavior or on a single resource depletion mechanism, this study integrates cognitive resource conservation responses and psychological resource depletion states within the framework of COR theory. Accordingly, the proposed model explains not only how teachers conserve their remaining resources by restricting cognitive exploration but also why the resources required for innovation may become insufficient, thereby providing a more comprehensive theoretical explanation of the association between perceived workload and TIB.

### 1.2 Conceptual background

#### 1.2.1 Perceived workload

Perceived workload refers to an individual’s subjective assessment of the amount, pace, and difficulty of work (Wang et al., 2025). It differs from objective workload because the same demands may be evaluated differently depending on the resources available to each individual (Liu et al., 2023). For university teachers, it reflects perceived demands arising from teaching, research, student supervision, administrative duties, and technological adaptation (Zhang, 2025).

#### 1.2.2 Teaching innovative behavior

TIB is the creation, promotion, and use of fresh and practical ideas in the classroom, including new course material, teaching strategies, learning exercises, and evaluation techniques (Cheng and Xu, 2025). Unlike teaching innovation ability, which concerns the capacity to innovate, TIB emphasizes observable actions through which innovative ideas are applied in practice.

#### 1.2.3 Need for cognitive closure

NFCC stands for the need for precise and unambiguous responses as well as the avoidance of ambiguity and confusion (Kruglanski and Boyatzi, 2012). Individuals with a stronger NFCC generally prefer order, predictability, and rapid decision-making (Ortega-Martín et al., 2021). Although relatively stable, this cognitive-motivational tendency may vary in its expression across situations (Büning et al., 2021).

#### 1.2.4 Thriving at work

According to Spreitzer et al. (2005), TW is a good psychological state marked by energy and learning. While learning represents the acquisition and application of information and abilities, vitality refers to feelings of energy at work. Unlike job satisfaction, thriving emphasizes employees’ simultaneous experience of energy and ongoing development (Goh et al., 2022).

### 1.3 Hypothesis development

#### 1.3.1 Perceived workload and teaching innovative behavior

University teachers undertake multiple responsibilities, including teaching preparation and delivery, research, student supervision, administrative duties, and professional development (Fuetterer et al., 2023; Liu et al., 2023). As these demands increase, teachers may perceive that their available time and energy are insufficient to meet their work requirements (Zhang, 2025). According to COR theory, individuals facing a threat of resource loss tend to protect their remaining resources and reduce their investment in activities requiring additional effort (Hobfoll, 1989). TIB is particularly resource intensive because generating and implementing new teaching ideas requires time, energy, cognitive flexibility, and a willingness to accept uncertain outcomes (Cheng and Xu, 2025). When teachers perceive a heavy workload, they may prioritize required duties and reduce their investment in innovative teaching practices (Huy et al., 2025). Workload and teachers’ innovation-related results have been found to be negatively correlated in earlier research (Huy et al., 2025; Li et al., 2025b). However, direct evidence concerning TIB among university teachers remains limited, as previous research has frequently focused on general creativity, innovative ability, or intrinsic motivation. Based on the preceding discussion, we hypothesize that:

*H1*: Perceived workload is negatively associated with TIB among university teachers.

#### 1.3.2 The mediating role of need for cognitive closure

From a COR perspective, when heavy work demands threaten teachers’ time and cognitive resources, seeking quick and definite solutions may help reduce further cognitive expenditure. Teachers experiencing high perceived workload may therefore rely more heavily on familiar structures and established approaches rather than consider multiple alternatives (Büning et al., 2021). A strong NFCC encourages rapid decision-making and reduces engagement with open-ended or uncertain processes (Wronska et al., 2019). Such a tendency may be incompatible with TIB, which requires openness, flexibility, and continued exploration. Previous studies have linked NFCC to lower creativity and innovation-related outcomes (Büning et al., 2021; Keem et al., 2023; Mao et al., 2022; Ortega-Martín et al., 2021). Keem et al. (2023) noted that the NFCC reduces creativity because it is a motivational tendency that encourages quick decision-making and avoids open-ended processes. Nevertheless, most studies have examined general creativity or innovative ability, leaving its association with university teachers’ actual innovative teaching practices insufficiently understood.

Drawing on COR theory, these relationships may be interpreted as a cognitive resource-conservation pathway. Higher perceived workload may place greater demands on university teachers’ limited time, energy, and cognitive resources and may be associated with a stronger preference for clear, stable, and predictable information, as reflected in higher NFCC. TIB, however, generally requires teachers to actively explore new information, tolerate uncertainty, and experiment with new teaching approaches. A stronger NFCC may therefore be associated with lower levels of TIB. Although the separate relationships between workload, NFCC, and innovation have received some attention, limited research has examined NFCC as an indirect pathway linking perceived workload with university teachers’ TIB. Based on the preceding discussion, we hypothesize that:

*H2:* Perceived workload is positively associated with NFCC among university teachers.

*H3*: NFCC is negatively associated with TIB among university teachers.

*H4*: Perceived workload is indirectly associated with lower TIB through a stronger NFCC among university teachers.

#### 1.3.3 The mediating role of thriving at work

TW is reflected in the simultaneous experience of vitality and learning (Spreitzer et al., 2005). From a COR perspective, vitality and learning represent psychological and developmental resources that may be diminished when work demands continuously consume employees’ energy and attention. Heavy perceived workload may reduce teachers’ vitality and restrict the time and energy available for learning and professional development (Wang et al., 2022). Workers who are vibrant and learning typically have greater enthusiasm and expertise for investigating and putting new concepts into practice (Islam and Idris, 2025). Existing studies have consistently associated TW with creativity and innovative behavior (Chen et al., 2024; Islam and Idris, 2025; Wang et al., 2022; Yang et al., 2021). For university teachers, vitality can support the effort required to develop new teaching practices, whereas learning provides the knowledge needed to improve teaching content and methods.

From a COR perspective, these relationships reflect psychological resource depletion, as individuals seek to conserve finite resources such as time, energy, and attention and are particularly sensitive to their loss (Hobfoll, 1989). Higher perceived workload may place sustained demands on university teachers’ psychological resources and may therefore be associated with lower levels of vitality and learning at work. When fewer resources are perceived to be available, teachers may also report less investment in acquiring new knowledge, exploring unfamiliar methods, and engaging in teaching reform. Although previous studies have established TW as an important correlate of innovation, its mediating role between perceived workload and TIB has received limited attention in the higher education context. In particular, few studies have examined this pathway alongside NFCC within a unified COR framework. Based on the preceding discussion, we hypothesize that:

*H5*: Perceived workload is negatively associated with TW among university teachers.

*H6*: TW is positively associated with TIB among university teachers.

*H7*: Perceived workload is indirectly associated with lower TIB through lower TW among university teachers.

#### 1.3.4 Gender differences

Previous studies suggest that male and female teachers may experience and respond to workload differently. Female teachers have frequently reported higher workload and role overload, partly because formal work responsibilities may coexist with additional caring and relational demands (Dethier et al., 2024; Koga et al., 2021; Zhou, 2023). These demands may create differences in the resources available for innovative teaching practices (Gunduz, 2021; Yao et al., 2024).

However, existing findings do not provide sufficiently consistent evidence to predict that the negative association between perceived workload and TIB is necessarily stronger for either gender. Moreover, gender differences in this relationship remain underexplored among university teachers. Therefore, a non-directional hypothesis is more appropriate:

*H8*: The association between perceived workload and TIB differs between male and female university teachers.

### 1.4 Current study

Drawing on COR theory, this study examines the association between perceived workload and TIB among university teachers. It further investigates the parallel mediating roles of NFCC and TW, representing cognitive resource-conservation and psychological resource-depletion pathways, respectively. In addition, the study examines whether the proposed structural relationships differ across gender groups. Figure 1 shows the proposed model.

**FIGURE 1:**
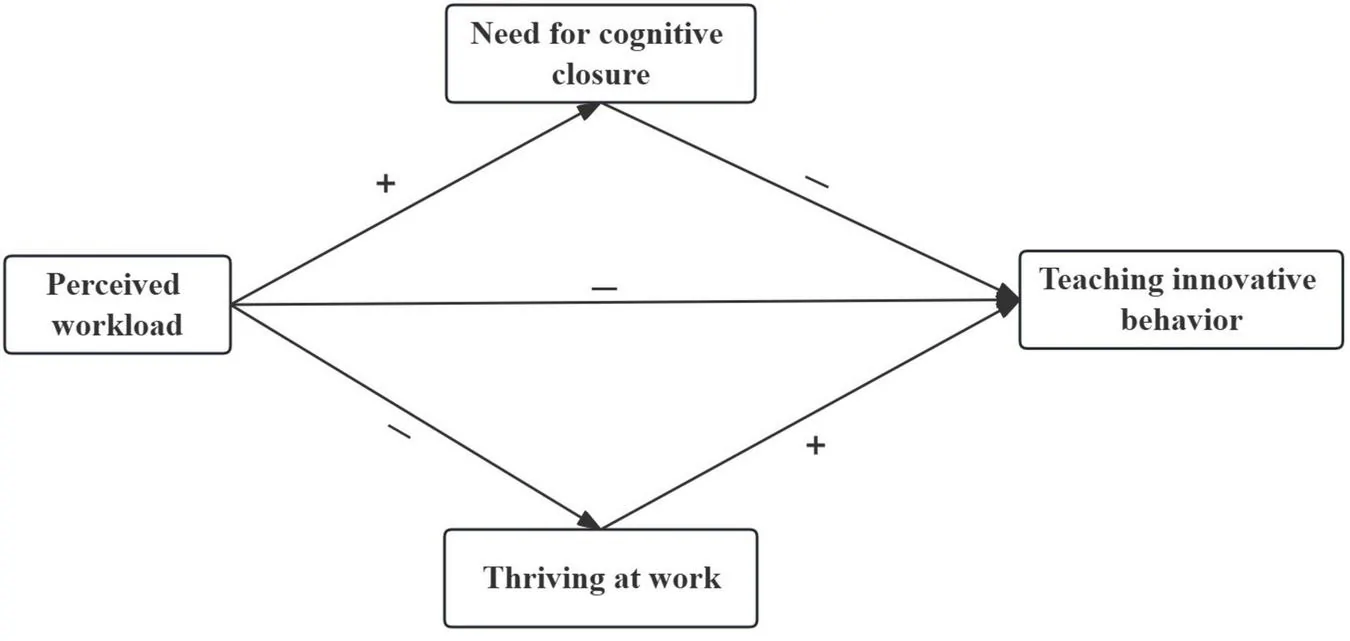
Research model.

## 2 Materials and methods

### 2.1 Participants

Data were collected from February to March 2026 using convenience sampling. Based on their accessibility and readiness to help with the dissemination of the questionnaire, the study team chose participating higher education institutions in China’s Henan region. To enhance the diversity of institutional representation, the selected institutions included both key universities and regular undergraduate institutions, although they were not selected randomly. Within each participating institution, university teachers were recruited with the assistance of relevant academic and administrative departments. Survey invitations containing a link to the online questionnaire were distributed through email, online communication platforms, and internal university networks. Eligible teachers who received the invitation could voluntarily choose whether to participate. The questionnaire was developed and administered through Questionnaire Star. Because the research team did not have access to a complete list of higher education institutions or university teachers, no sampling frame was available for probability sampling. Neither the participating institutions nor the individual teachers were selected randomly. Therefore, although data collection involved two operational levels, institutional selection and teacher recruitment, this procedure did not constitute multistage probability sampling. Instead, the entire sampling process was based on non-probability convenience sampling. After being told about the goal of the study, voluntary participation, data confidentiality, and data usage, all participants gave their informed consent.

As per to sample analysis method established by Kline (2018), every polling issue needs answers from minimum ten respondents. Consumption form for this inquiry contains a total of 40 elements. With a 20% attrition rate, the total sample number is 480 persons. To fulfill study needs, 660 questionnaires were circulated, with 650 returned.

During data screening, 16 questionnaires were excluded because they either contained excessive missing data or showed evidence of response-pattern bias. Specifically, questionnaires were considered invalid when more than 20% of the items were unanswered or when respondents selected the same extreme response option for at least 80% of the items, as such response patterns may reduce the accuracy of the measurement results (Wang et al., 2012). Ultimately, 634 valid questionnaires were retained for analysis, including responses from 273 male and 361 female teachers (see Table 1).

**TABLE 1:** Demographic characteristics.

Category	Subcategory	Number	Percentage
Gender	Male	273	43.1%
	Female	361	56.9%
Teaching experience	0–3 years	133	21.0%
	4–7 years	237	37.4%
	8–15 years	120	18.9%
	16–25 years	144	22.7%
Educational background	Bachelor’s	23	3.6%
	Master’s	342	53.9%
	Doctorate	214	33.8%
	Postdoc	55	8.7%
Academic rank	Lecturer	357	56.3%
	Associate professor	188	29.7%
	Professor	89	14.0%

### 2.2 Measures

#### 2.2.1 Perceived workload

The perceived workload of teachers at universities was assessed using the Workload Scale (ECT) (Cruz et al., 2018). It has six items and uses a 5-point rating system from 1 (never) to 5 (very frequently). Higher ratings imply a larger perceived workload among participants. This scale has been proven to be valid among the Chinese population (Wang, 2024). The Cronbach’s α for the full scale was 0.860, indicating good reliability.

#### 2.2.2 Need for cognitive closure

The Need for Closure Scale was used to assess the NFCC of university teachers (Webster and Kruglanski, 1994). This scale has 15 items with a 6-point rating system. Higher scores indicate a greater level of the NFCC among participants. This scale has been proven to be valid among the Chinese population (Hang et al., 2024). In the current investigation, the scale showed outstanding internal consistency (Cronbach’s α = 0.942).

#### 2.2.3 Thriving at work

TW is measured using the Chinese version of the 10-item scale (Porath et al., 2012). This scale has demonstrated excellent measurement performance within the Chinese cultural context (He et al., 2023). The scale contains 10 items, of which items 4 and 8 are scored in reverse. The scale has 5-point rating system. Higher marks show higher levels of TW for respondents. The Cronbach’s α for this scale was 0.926, suggesting solid reliability.

#### 2.2.4 Teaching innovative behavior

TIB was measured with the four items reported by Chen et al. (2025), originally derived from the Employee Innovative Behavior Scale developed by Scott and Bruce (1994). The original four-item structure was retained, with only the item referents adapted to the university teaching context; the intended meanings and response format remained unchanged. The adapted measure was reviewed by three experts and pilot-tested with 30 university teachers, confirming its clarity and contextual appropriateness. The four items assessed the identification of teaching problems, generation of teaching ideas, exploration of teaching methods, and development of creative teaching solutions. Responses were recorded on a five-point scale ranging from 1 (completely disagree) to 5 (completely agree), with higher scores indicating stronger TIB. The measure demonstrated satisfactory internal consistency (Cronbach’s α = 0.773).

### 2.3 Data analysis

SmartPLS 4 was used to carry out partial least squares structural equation modeling (PLS-SEM). PLS-SEM was chosen because the study examined several direct and indirect correlations in addition to the model’s explanatory power for the endogenous variables and its out-of-sample prediction performance. The measurement model was assessed for reliability and validity, common method bias, and collinearity, followed by structural model evaluation using path coefficients, *R*^2^, *f*^2^, and predictive performance. Direct and indirect associations were tested using 5,000 bootstrap resamples with 95% confidence intervals, while out-of-sample predictive power was assessed using PLSpredict. Because PLS-SEM was used as the primary analytical approach, global fit indices associated with covariance-based structural equation modeling, such as CFI, TLI, and RMSEA, were not reported.

## 3 Results

### 3.1 Descriptive statistics and correlation analysis

To fully understand the central tendency and distribution characteristics of each latent variable, Table 2 reports its mean (M), standard deviation (SD), skewness (SK), and kurtosis (Kur), respectively. The results indicate that the SK and Kur of each variable fall inside appropriate limits, and the data generally satisfy the assumption of normal distribution (Kline, 2018).

**TABLE 2:** Correlation analysis of study variables.

Variable	M ± SD	*SK*	*Kur*	PW	TIB	NFCC	TW
PW	2.883 ± 0.830	0.440	–0.060	1	1	1	1
TIB	3.806 ± 0.796	–1.604	1.936	–0.573***			
NFCC	2.137 ± 0.749	2.259	4.948	0.519***	–0.599***		
TW	3.565 ± 0.833	–1.086	0.428	–0.469***	0.587***	–0.536***	

*N* = 634; ***p < 0.001; PW, Perceived Workload; TW, Thriving at Work; NFCC, Need for Cognitive Closure; TIB, Teaching Innovative Behavior.

Furthermore, we found significant correlations between perceived workload, NFCC, TW, and TIB (*p* < 0.001, *N* = 634). Specifically, perceived workload showed a significant negative correlation with TIB (*r* = –0.573, *p* < 0.001) and TW (*r* = –0.469, *p* < 0.001), while exhibiting a significant positive correlation with NFCC (*r* = 0.519, *p* < 0.001). TIB was significantly negatively correlated with NFCC (*r* = –0.599, *p* < 0.001) and positively correlated with TW (*r* = 0.587, *p* < 0.001). These correlation results provided preliminary support for H1, H2, H3, H5, and H6, which were subsequently tested using the structural model.

### 3.2 Measurement model

The reflective measurement model demonstrated adequate reliability and validity. Indicators with loadings above 0.60 were retained when CR and AVE met the recommended thresholds and theoretical relevance was maintained. As shown in Table 3, indicator reliability ranged from 0.388 to 0.681. Indicators with reliability values below 0.50 were retained because their outer loadings exceeded 0.60, the corresponding constructs met the recommended CR and AVE thresholds, and the indicators were considered theoretically meaningful. Outer VIF values ranged from 1.394 to 2.691, indicating no serious collinearity. Cronbach’s α, CR, and AVE exceeded 0.70, 0.80, and 0.50, respectively, supporting reliability and convergent validity (Hair et al., 2021). All indicators also loaded highest on their intended constructs, further supporting discriminant validity.

**TABLE 3:** Reliability and validity assessment.

Variable	Items	Outer loadings	Indicator reliability	Cronbach’s α	CR	AVE
TIB	TIB1	0.825	0.681	0.773	0.855	0.596
	TIB2	0.782	0.612			
	TIB3	0.783	0.613			
	TIB4	0.692	0.479			
NFCC	NFCC1	0.731	0.534	0.942	0.949	0.555
	NFCC2	0.751	0.564			
	NFCC3	0.786	0.618			
	NFCC4	0.764	0.584			
	NFCC5	0.726	0.527			
	NFCC6	0.798	0.637			
	NFCC7	0.705	0.497			
	NFCC8	0.733	0.537			
	NFCC9	0.780	0.608			
	NFCC10	0.812	0.659			
	NFCC11	0.623	0.388			
	NFCC12	0.751	0.564			
	NFCC13	0.739	0.546			
	NFCC14	0.734	0.539			
	NFCC15	0.721	0.520			
PW	PW1	0.713	0.508	0.860	0.895	0.588
	PW2	0.759	0.576			
	PW3	0.776	0.602			
	PW4	0.785	0.616			
	PW5	0.784	0.615			
	PW6	0.784	0.615			
TW	TW1	0.788	0.621	0.926	0.937	0.600
	TW2	0.747	0.558			
	TW3	0.789	0.623			
	TW4	0.803	0.645			
	TW5	0.798	0.637			
	TW6	0.775	0.601			
	TW7	0.730	0.533			
	TW8	0.766	0.587			
	TW9	0.765	0.585			
	TW10	0.782	0.612			

CR, Composite Reliability; AVE, Average Variance Extracted; PW, Perceived Workload; TW, Thriving at Work; NFCC, Need for Cognitive Closure; TIB, Teaching innovative behavior.

Discriminant validity was examined through cross-loadings, the Fornell–Larcker criterion, and the heterotrait–monotrait ratio (HTMT) (Hair et al., 2021). As reported in Supplementary Table 1, each indicator loaded most strongly on its assigned construct. Table 4 further shows that the diagonal square roots of AVE exceeded the corresponding interconstruct correlations (Fornell and Larcker, 1981). In addition, the HTMT ratios in Table 5 were all below 0.85 (Henseler et al., 2015). Collectively, these results confirmed adequate discriminant validity.

**TABLE 4:** Fornell-Larcker criterion.

Variable	TIB	NFCC	PW	TW
TIB	**0.772**	**0.745**	**0.767**	**0.775**
NFCC	–0.601			
PW	–0.579	0.527		
TW	0.591	–0.539	–0.477	

The value in bold on the diagonal is the square root of the AVE; PW = Perceived Workload; TW, Thriving at Work; NFCC, Need for Cognitive Closure; TIB, Teaching Innovative Behavior.

**TABLE 5:** Heterotrait-monotrait ratio.

Construct pair	HTMT	2.5%	97.5%	Conclusion
PW <–> NFCC	0.576	0.470	0.658	Supported
TIB <–> NFCC	0.697	0.589	0.788	Supported
TIB <–> PW	0.703	0.632	0.763	Supported
TW <–> NFCC	0.569	0.476	0.651	Supported
TW <–> PW	0.524	0.434	0.606	Supported
TW <–> TIB	0.691	0.603	0.768	Supported

PW, Perceived Workload; TW, Thriving at Work; NFCC, Need for Cognitive Closure; TIB, Teaching innovative behavior.

### 3.3 Structural model

#### 3.3.1 Common method bias test

Potential common method bias was evaluated using Harman’s single-factor test and full collinearity assessment. The unrotated analysis extracted four factors, with the largest accounting for 39.477% of the variance, below the recommended 40% criterion (Podsakoff and Organ, 1986).

In addition, full collinearity VIFs ranged from 1.517 to 1.901, remaining below the 3.3 cutoff proposed by Kock (2015). Together, these results suggest that common method bias was unlikely to substantially affect the findings.

#### 3.3.2 Collinearity test

Predictor collinearity was examined using VIF values. As reported in Table 6, the inner VIF values ranged from 1.000 to 1.626, all below the recommended threshold of 3.3, indicating no serious collinearity in the structural model (Hair et al., 2021).

**TABLE 6:** Collinearity test of the structural model.

Variable	TIB	NFCC	PW	TW
TIB				
NFCC	1.626			
PW	1.493	1.000		1.000
TW	1.519			

PW, Perceived Workload; TW, Thriving at Work; NFCC, Need for Cognitive Closure; TIB, Teaching innovative behavior.

#### 3.3.3 Path hypothesis

Figure 2 and Table 7 show that perceived workload (β = –0.283, *t* = 7.048, *p* < 0.001, *f*^2^ 0.111) and NFCC (β = –0.290, *t* = 5.674, *p* < 0.001, *f*^2^ 0.107) were negatively associated with TIB, whereas TW was positively associated with TIB (β = 0.300, *t* = 6.964, *p* < 0.001, *f*^2^ 0.122). Perceived workload was positively associated with NFCC (β = 0.527, *t* = 12.214, *p* < 0.001, *f*^2^ 0.385) and negatively associated with TW (β = –0.477, *t* = 12.119, *p* < 0.001, *f*^2^ 0.294). Thus, H1, H2, H3, H5, and H6 were supported. The effect sizes were large for PW → NFCC, medium for PW → TW, and small for the remaining three paths.

**FIGURE 2:**
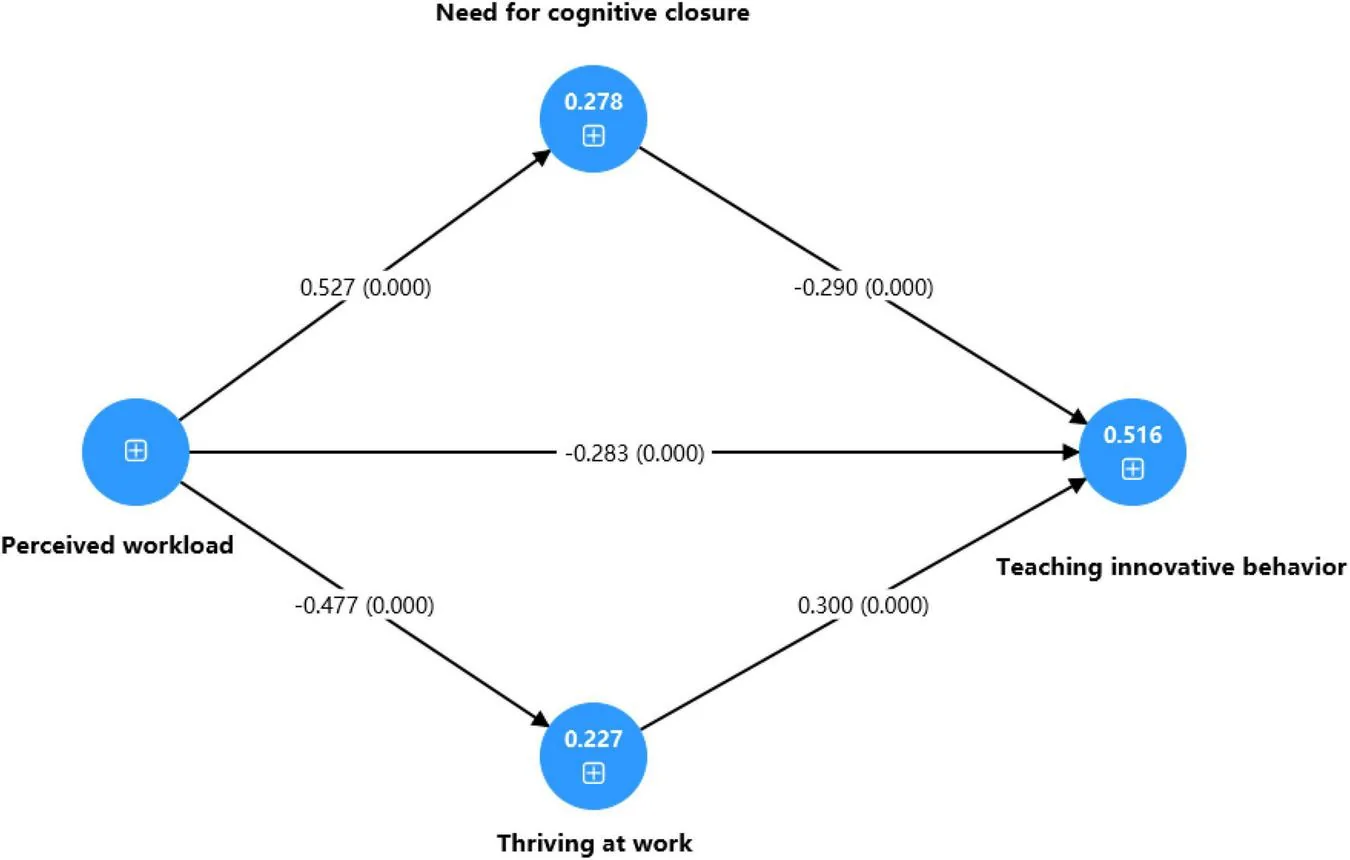
Mediating effect model.

**TABLE 7:** Path hypothesis testing results.

Path	β	*t*	*p*	*f* ^2^	95% CI	Result
NFCC → TIB	–0.290	5.674	< 0.001	0.107	[–0.393, –0.193]	Supported
PW → TIB	–0.283	7.048	< 0.001	0.111	[–0.359, –0.200]	Supported
PW → NFCC	0.527	12.214	< 0.001	0.385	[0.437, 0.608]	Supported
PW → TW	–0.477	12.119	< 0.001	0.294	[–0.551, –0.397]	Supported
TW → TIB	0.300	6.964	< 0.001	0.122	[0.218, 0.386]	Supported

PW, Perceived Workload; TW, Thriving at Work; NFCC, Need for Cognitive Closure; TIB, Teaching Innovative Behavior; *f*^2^ values of 0.02, 0.15, and 0.35 indicate small, medium, and large effect sizes, respectively.

#### 3.3.4 *R*^2^ and *Q*^2^ of the structural model

The structural model was assessed using *R*^2^ and Stone–Geisser’s Q^2^. As reported in Table 8, the model accounted for 51.6% of the variance in TIB (*R*^2^ = 0.516), 27.8% in NFCC (*R*^2^ = 0.278), and 22.7% in TW (*R*^2^ = 0.227). Their respective Q^2^ values were 0.332, 0.272, and 0.223. All values exceeded zero, confirming predictive relevance for the three endogenous constructs (Hair et al., 2023). Overall, the model showed moderate explanatory power for TIB but relatively limited explanatory power for NFCC and TW.

**TABLE 8:** Predictive relevance of the structural model.

Constructs	*R* ^2^	*Q* ^2^
TIB	0.516	0.332
NFCC	0.278	0.272
TW	0.227	0.223

TW, Thriving at Work; NFCC, Need for Cognitive Closure; TIB, Teaching Innovative Behavior.

#### 3.3.5 Predictive assessment using PLSpredict

Out-of-sample prediction was evaluated using PLSpredict with 10 folds and 10 repetitions. The *Q*^2^ predict values ranged from 0.092 to 0.227 and were all positive, demonstrating predictive relevance against the indicator-average benchmark. However, PLS-SEM produced lower RMSE values than the linear model for only 3 of the 29 indicators, indicating limited predictive power relative to this benchmark. These findings suggest that the model provides greater explanatory than predictive utility.

### 3.4 Mediation effect analysis

Bootstrapping with 5,000 resamples was used to examine the indirect associations of perceived workload with TIB through NFCC and TW (Nitzl et al., 2016).

As shown in Table 9, the indirect association through NFCC was significant (β = –0.153, *t* = 4.559, *p* < 0.001, 95% CI [–0.224, –0.094]), supporting H4. The indirect association through TW was also significant (β = –0.143, *t* = 5.659, *p* < 0.001, 95% CI [–0.196, –0.098]), supporting H7. Because neither confidence interval included zero, both indirect associations were statistically significant.

**TABLE 9:** Mediating effect analysis.

Path	β	*T-*value	*P-*value	95% CI	Result
PW → NFCC → TIB	–0.153	4.559	< 0.001	[–0.224, –0.094]	Supported
PW → TW → TIB	–0.143	5.659	< 0.001	[–0.196, –0.098]	Supported

PW, Perceived Workload; TW, Thriving at Work; NFCC, Need for Cognitive Closure; TIB, Teaching Innovative Behavior.

### 3.5 Multigroup analysis

#### 3.5.1 Measurement invariance analysis

To compare differences in route coefficients between genders, this study used the multigroup PLS analysis approach. This strategy has been frequently used in the current literature (Al-Awlaqi et al., 2023; Huegel and Kreutzer, 2020). PLS multigroup analysis was used to compare structural path coefficients between male and female teachers. Before the group comparison, measurement invariance was examined using the MICOM procedure (Henseler et al., 2016). Configural invariance was supported because both groups used the same indicators, data treatment, algorithm settings, and model specifications.

MICOM Step 2 supported compositional invariance for all constructs, as the original correlations met or exceeded the corresponding 5% quantiles and all permutation *p*-values were above 0.05 (Table 10; Hair et al., 2019). Together with the establishment of configural invariance in Step 1, these results supported partial measurement invariance across the gender groups, thereby permitting the subsequent multigroup analysis.

**TABLE 10:** MICOM step 2: compositional invariance across male and female groups.

Construct	Original correlation	5% quantile of empirical distribution of c_u_	*P*-value	Compositional invariance
NFCC	1.000	0.999	0.683	Yes
PW	0.999	0.999	0.163	Yes
TIB	0.998	0.998	0.056	Yes
TW	0.999	0.999	0.065	Yes

PW, Perceived Workload; TW, Thriving at Work; NFCC, Need for Cognitive Closure; TIB, Teaching Innovative Behavior.

#### 3.5.2 Multigroup analysis

After partial measurement invariance had been established, a multigroup analysis was conducted to examine whether the association between perceived workload and TIB differed across gender groups. As shown in Table 11, the path coefficient for perceived workload and TIB differed significantly between male and female teachers (*Δβ* = 0.177, *t* = 2.168, *p* = 0.030). Specifically, the negative association was stronger in the female group (β = –0.353) than in the male group (β = –0.176). Therefore, H8 was supported. However, this group difference should not be interpreted as evidence of inherent gender differences in workload sensitivity or coping styles. Because the study did not directly measure the individual and contextual factors underlying this difference, its explanation requires further investigation.

**TABLE 11:** Multigroup path analysis (male and female).

Construct	β (Male)	β (Female)	*Δβ* (male - Female)	*T-*value (male vs. female)	*P-*value (male vs. female)
PW → TIB	–0.176	–0.353	0.177	2.168	0.030
NFCC → TIB	–0.315	–0.274	–0.041	0.394	0.694
PW → NFCC	0.572	0.488	0.084	0.950	0.343
PW → TW	–0.541	–0.427	–0.115	1.440	0.150
TW → TIB	0.391	0.236	0.155	1.746	0.081

PW, Perceived Workload; TW, Thriving at Work; NFCC, Need for Cognitive Closure; TIB, Teaching Innovative Behavior.

## 4 Discussion

This study examined the association between perceived workload and university teachers’ TIB, as well as the mediating roles of NFCC and TW, and further conducted a gender-based multigroup analysis. All hypotheses were supported. Higher perceived workload was associated with lower TIB, both directly and indirectly through higher NFCC and lower TW. This negative association was stronger among female teachers than among male teachers.

There was a significant negative association between perceived workload and university teachers’ TIB, supporting H1. This finding is consistent with Huy et al. (2025), who examined the job demands, including workload and time pressure, of 1,071 Vietnamese teachers and reported a negative association between workload and teachers’ innovation-related outcomes. In the present study, higher perceived workload may be accompanied by greater exhaustion and stress, leaving teachers with fewer resources and less motivation to engage in innovative teaching practices (Li et al., 2025a). Furthermore, this finding extends our understanding of teacher wellbeing by suggesting that perceived workload is associated not only with mental health but also with innovative behaviors closely related to educational quality. These results emphasize how crucial institutional solutions are for controlling excessive workload and safeguarding the resources needed for TIB.

Perceived workload was significantly positively associated with NFCC, whereas NFCC was significantly negatively associated with TIB. Perceived workload was also indirectly associated with lower TIB through a stronger NFCC, supporting H2, H3, and H4. Although previous studies have not directly examined this complete indirect pathway, existing evidence provides theoretical support for its component relationships. First, the present findings are consistent with previous studies linking a stronger NFCC to lower creativity and innovation-related outcomes (Keem et al., 2023; Mao et al., 2022). Second, from the perspective of COR theory (Hobfoll, 1989), high perceived workload may place pressure on university teachers’ limited cognitive resources and be associated with a stronger preference for certainty, structure, and familiar teaching practices. Such a cognitive orientation is inconsistent with the openness, flexibility, and exploration required for TIB (Wronska et al., 2019). Therefore, the findings connect these previously separate research strands by identifying NFCC as a potential cognitive resource-conservation pathway linking perceived workload with lower TIB. Beyond their obvious correlation, this offers a more complex explanation of the link between workload and creative teaching methods.

Perceived workload was significantly negatively associated with TW, whereas TW was significantly positively associated with TIB. Perceived workload was also indirectly associated with lower TIB through lower TW, supporting H5, H6, and H7. This result is in line with other research showing a favorable correlation between TW and results connected to innovation (Chen et al., 2024; Islam and Idris, 2025). From a COR perspective, perceived workload may consume teachers’ energy and limit their opportunities for learning, resulting in lower levels of TW. Because TW is characterized by the joint experience of vitality and learning (Spreitzer et al., 2005), lower thriving may be associated with fewer psychological and developmental resources available for exploring and implementing new teaching practices. Therefore, TW represents a potential psychological resource-depletion pathway linking perceived workload with lower TIB. This finding extends previous research that has primarily examined the direct relationship between workload and innovation-related outcomes by identifying an additional indirect pathway among university teachers.

The multigroup analysis showed that the association between perceived workload and TIB differed significantly between male and female teachers. Specifically, the negative association was stronger among female teachers than among male teachers, supporting H8. This result is mostly in line with other research showing that female educators may report greater workloads, more emotional labor, and expectations from numerous roles (Dethier et al., 2024; Sanabrias-Moreno et al., 2023). From a COR perspective, such additional demands may be associated with greater pressure on the time, energy, and psychological resources available for innovative teaching practices (Hobfoll, 1989; Yao et al., 2024). Nevertheless, the present study did not directly measure gender-role expectations, emotional labor, or coping strategies. Therefore, these factors should be regarded as possible contextual explanations rather than established mechanisms underlying the observed gender difference. Future research should directly examine these factors to clarify why the association between perceived workload and TIB may vary across gender groups.

## 5 Research implications

This study examined the association between perceived workload and university teachers’ TIB by constructing a parallel mediation model involving NFCC and TW. It also examined whether the proposed relationships differed across gender groups. The results offer theoretical and practical insights into the processes connected to university teacher TIB that are related to cognitive and psychological resources.

### 5.1 Theoretical implications

First, this study advances the understanding of workload and innovation by clarifying the mechanisms linking perceived workload to TIB. Existing research has largely examined general creative or innovative outcomes, leaving the processes underlying university teachers’ TIB insufficiently understood. The significant indirect associations through NFCC and TW indicate that perceived workload is associated with TIB through both teachers’ tendency to seek cognitive certainty and the availability of psychological resources for innovation. These findings offer a dual-process account of how work demands relate to innovative teaching.

Second, this study extends the explanation offered by COR theory for the association between perceived workload and TIB by distinguishing and integrating two complementary resource pathways within a parallel mediation model. NFCC represents a cognitive resource conservation pathway, whereby teachers conserve their remaining cognitive resources by developing a stronger preference for certainty, relying on familiar teaching practices, and reducing further exploration. TW represents a psychological resource depletion pathway, reflecting a lower level of the resources required for teachers to engage in teaching innovation. The two pathways, respectively, reveal how teachers protect their remaining resources under resource pressure and whether they still possess the resources necessary for innovation; therefore, neither can substitute for the other. This study integrates cognitive resource conservation responses and the availability of psychological resources into a single framework, in contrast to earlier research that has mostly concentrated on the direct relationship between workload and innovative behavior or on a single resource depletion process. In doing so, it extends the explanatory scope of COR theory from whether resources are diminished to how individuals respond to resource pressure. A more complex dual-pathway explanation of the relationship between perceived workload and TIB is offered by this integration.

Third, the results improve how COR theory is applied to higher education instruction. Rather than treating resource loss as a single undifferentiated process, this study distinguishes between a cognitive tendency to conserve remaining resources and the reduced availability of positive psychological and developmental resources. The findings suggest that perceived workload is associated with innovative teaching not only through the resources available to teachers but also through how teachers may allocate and protect those resources under pressure. In addition, the stronger negative association between perceived workload and TIB among female teachers identifies gender as a potential boundary condition, suggesting that the relationship between resource pressure and innovative teaching may vary across teacher groups and warrants further investigation.

### 5.2 Practical implications

At the individual level, the findings suggest that university teachers may benefit from protecting the cognitive and psychological resources required for TIB. When experiencing a heavy workload, teachers could remain aware of an increased preference for certainty and familiar routines and intentionally maintain openness to alternative teaching approaches. Teachers may also support their TW by setting achievable teaching goals, participating in professional exchanges, seeking peer feedback, and reflecting on their innovative teaching practices. These activities may help preserve vitality and learning opportunities under demanding work conditions. The observed gender difference suggests that workload-management and resource-support strategies may need to be sensitive to the different work and life circumstances experienced by male and female teachers. However, these strategies should be implemented on the basis of each teacher’s actual workload, available resources, role demands, and personal circumstances rather than gender alone.

At the institutional level, universities should create working conditions that support TIB. First, institutions could regularly assess faculty workload, improve the balance among teaching, research, and administrative responsibilities, and simplify unnecessary administrative procedures. Second, teaching innovation workshops, teaching reform projects, exemplary cases, and practical guidance could provide teachers with useful reference points and reduce the cognitive demands involved in exploring unfamiliar teaching approaches. Third, universities could establish teacher learning communities, provide continuous professional development opportunities, and strengthen peer and organizational support to promote teachers’ vitality and learning. Evaluation and incentive systems should also give appropriate recognition to teachers’ efforts to develop and implement innovative teaching practices.

At the policy level, teacher workload management and support for TIB could be incorporated into higher education quality assurance and faculty development systems. Education authorities should encourage universities to avoid excessive accumulation of work demands that may constrain the resources available for innovative teaching. Faculty training programs could include content on cognitive flexibility, resource management, and TW to help teachers better understand the psychological conditions associated with innovative teaching practices. Policymakers could also support dedicated funding, professional development platforms, and institutional environments that encourage responsible experimentation and tolerate reasonable failure in teaching innovation.

## 6 Limitations and future research directions

This study has several limitations. First, all variables were measured using self-reported questionnaires, which may be affected by response subjectivity and may not fully capture teachers’ actual innovative teaching practices. Although the adapted TIB scale demonstrated acceptable reliability, future studies could combine self-reports with classroom observations, interviews, peer evaluations, or teaching records to obtain a more comprehensive assessment.

Second, the cross-sectional design does not establish the temporal or causal ordering of perceived workload, NFCC, TW, and TIB. The mediation findings represent statistical indirect associations rather than causal mechanisms. Longitudinal, multiwave, or experimental studies are needed to clarify how these relationships unfold over time.

Finally, the model demonstrated limited out-of-sample predictive power relative to the linear model benchmark. Future research should include additional theoretically relevant predictors and test the model across more diverse institutional and regional samples to improve its generalizability and predictive performance.

## 7 Conclusion

Using data from 634 university teachers, this study tested a structural equation model examining the association between perceived workload and TIB, the parallel mediating roles of NFCC and TW, and gender differences in the proposed relationships. The results showed that perceived workload was significantly negatively associated with TIB. Perceived workload was also indirectly associated with lower TIB through a stronger NFCC and lower TW. These findings indicate two conceptually distinct but directionally consistent indirect pathways: a cognitive resource-conservation pathway through NFCC and a psychological resource-depletion pathway through TW. Furthermore, the negative association between perceived workload and TIB was stronger among female teachers than among male teachers. Drawing on COR theory, this study provides an integrated framework for understanding the cognitive and psychological resource-related processes associated with university teachers’ TIB. The findings also offer practical insights for higher education institutions seeking to manage faculty workload, promote cognitive flexibility, and support teachers’ vitality and learning. Future studies should use larger, more diverse samples and longitudinal designs while considering additional individual and organizational correlates of university teachers’ TIB.

## Data Availability

The raw data supporting the conclusions of this article will be made available by the authors, without undue reservation.
