# Supplementary material for: Perceived workload and teaching innovative behavior in university teachers: parallel mediation by need for cognitive closure and thriving at work, gender multi-group
Source: Front Psychol. 2026 Sep 14;17:1837443. doi: 10.3389/fpsyg.2026.1837443 (PMC13616634; doi:10.3389/fpsyg.2026.1837443)
Supplement: Supplementary file 1 [file Supplementary_file_1.docx]

**Supplementary Material**

**Manuscript title:**

Perceived Workload and Teaching Innovative Behavior in University Teachers: Parallel Mediation by Need for Cognitive Closure and Thriving at work, Gender Multi-Group

Table S1. Cross-loadings of the measurement model

| **Item** | **TIB** | **NFCC** | **PW** | **TW** |
| --- | --- | --- | --- | --- |
| TIB1 | **0.825** | -0.502 | -0.441 | 0.508 |
| TIB2 | **0.782** | -0.480 | -0.463 | 0.456 |
| TIB3 | **0.783** | -0.475 | -0.480 | 0.465 |
| TIB4 | **0.692** | -0.391 | -0.400 | 0.390 |
| NFCC1 | -0.375 | **0.731** | 0.421 | -0.384 |
| NFCC2 | -0.494 | **0.751** | 0.424 | -0.397 |
| NFCC3 | -0.489 | **0.786** | 0.446 | -0.419 |
| NFCC4 | -0.508 | **0.764** | 0.402 | -0.416 |
| NFCC5 | -0.420 | **0.726** | 0.397 | -0.443 |
| NFCC6 | -0.488 | **0.798** | 0.378 | -0.415 |
| NFCC7 | -0.506 | **0.705** | 0.402 | -0.444 |
| NFCC8 | -0.370 | **0.733** | 0.332 | -0.362 |
| NFCC9 | -0.435 | **0.780** | 0.396 | -0.408 |
| NFCC10 | -0.507 | **0.812** | 0.453 | -0.446 |
| NFCC11 | -0.361 | **0.623** | 0.324 | -0.298 |
| NFCC12 | -0.422 | **0.751** | 0.415 | -0.415 |
| NFCC13 | -0.407 | **0.739** | 0.325 | -0.382 |
| NFCC14 | -0.422 | **0.734** | 0.371 | -0.366 |
| NFCC15 | -0.456 | **0.721** | 0.366 | -0.397 |
| PW1 | -0.465 | 0.413 | **0.713** | -0.349 |
| PW2 | -0.401 | 0.337 | **0.759** | -0.281 |
| PW3 | -0.430 | 0.337 | **0.776** | -0.360 |
| PW4 | -0.429 | 0.451 | **0.785** | -0.343 |
| PW5 | -0.403 | 0.390 | **0.784** | -0.378 |
| PW6 | -0.513 | 0.468 | **0.784** | -0.455 |
| TW1 | 0.507 | -0.438 | -0.400 | **0.788** |
| TW2 | 0.438 | -0.439 | -0.398 | **0.747** |
| TW3 | 0.457 | -0.406 | -0.333 | **0.789** |
| TW4 | 0.479 | -0.450 | -0.410 | **0.803** |
| TW5 | 0.453 | -0.424 | -0.365 | **0.798** |
| TW6 | 0.506 | -0.462 | -0.373 | **0.775** |
| TW7 | 0.389 | -0.337 | -0.330 | **0.730** |
| TW8 | 0.445 | -0.429 | -0.356 | **0.766** |
| TW9 | 0.357 | -0.292 | -0.311 | **0.765** |
| TW10 | 0.511 | -0.457 | -0.392 | **0.782** |

**Note:** Bold values indicate the loadings of the indicators on their intended constructs. NFCC = Need for Cognitive Closure; PW = Perceived Workload; TIB = Teaching Innovative Behavior; TW = Thriving at Work.
